# Prior Knowledge Norms for Naming Country Outlines: An Open Stimulus Set

**DOI:** 10.5334/joc.260

**Published:** 2023-01-30

**Authors:** Thomas Wilschut, Maarten van der Velde, Florian Sense, Hedderik van Rijn

**Affiliations:** 1 Department of Experimental Psychology, University of Groningen, NL

**Keywords:** Learning, Stimulus development, Response accuracy, Response speed, Long-term memory

## Abstract

Paired-associate stimuli are an important tool in learning and memory research. In cognitive psychology, many studies use materials of which the learners are expected to have little to no prior knowledge. Despite their theoretical usefulness, conclusions from these studies are difficult to generalize to real-world learning contexts, where learners can be expected to have varying degrees of prior knowledge. Here, we present an ecologically valid stimulus set with 112 country outline-name pairs, and report response times and prior knowledge for these items in 285 largely Western European participants. Prior knowledge per item ranged from very high (94.4%) to zero (0.3%), thereby allowing researchers to select materials of which participants can be expected to have any given amount of prior knowledge. As such, this database provides a useful tool for research on real-world learning. The database can be accessed at: https://osf.io/q25rd/.

## Introduction

Most experiments that examine the mechanisms of learning and forgetting of declarative information use stimulus sets of which learners are expected to have little to no prior knowledge. For example, pairs of non-words (e.g., Gupta et al., 2004), pairs of words that are semantically (weakly) related (e.g., Kornell, Hays, & Bjork, 2009), foreign language-English word pairs (e.g., see Grimaldi, Pyc, & Rawson, 2010; Nelson, & Dunlosky, 1994) or nonsensical pictures (e.g., Gauthier & Tarr, 1997; Nishimoto, Ueda, Miyawaki, Une & Takahashi, 2010) are frequently used in experimental settings. Using these materials comes with a clear advantage: The performance of the participants is unlikely to be biased by their background (e.g., their educational history or prior knowledge) which allows researchers to easily compare learning rates across individuals with similar starting points. Because of the popularity of the above-mentioned materials in learning research, various stimulus sets with normative performance data are available to researchers in the field.

Outside the laboratory, however, learning rarely occurs without prior knowledge. In fact, prior knowledge is generally considered to be amongst the most important factors predicting learning outcomes (Dochy, De Ridjy, & Dyck, 2002; Hailikari, Nevgi, & Lindblom-Ylänne, 2007; Portier, & Wagemans, 1995; Pressley, & McCormick, 1995; Thompson, & Zamboanga, 2003). Prior knowledge has been shown to positively influence both acquisition and retrieval of various types of study materials (see Dochy, Segers, & Buehl, 1999, for a review) and the capacity to apply newly acquired knowledge to higher-order problem solving tasks (Dresel, Ziegler, Broome, & Heller, 1998; Nathanso, Paulhus, & Williams, 2004). Because of the importance of prior knowledge in educational practice, the usefulness of materials of which participants have no prior knowledge is limited.

In this study, we tested prior knowledge of a set of country outline-name paired associates, in order to create a reference stimulus set that can be used by learning researchers in various areas in cognitive science (for a related data set on geography learning in an adaptive learning context, see Papoušek, Pelánek, & Stanislav, 2016). There are numerous advantages to using country outline-name paired associates as stimuli in a learning experiment. First, participants can reasonably be expected to have (varying degrees of) prior knowledge on the topic, as (world country) toponymy is commonly taught in elementary school. Second, learning country names from their outline requires the learner to integrate already familiar information (e.g., knowledge about the country) to new information such as the characteristics of the country outline. This makes these materials suitable in studies investigating real-world learning problems, where the integration of old and new knowledge is very common. Third, on a more practical level, the current 112-item stimulus set can easily be extended to a larger set by adding other countries, states, areas, city locations, or other topographical features. Finally, unlike word pairs, studying country outlines is relatively independent of the participants’ native language (As further elaborated in the discussion section below, prior knowledge of toponymy items is not independent of the learners’ geographical location, which on one hand limits the universal applicability of the materials, but also facilitates the utilization of within-item differences in experimental settings).

## Methods

### Participants

In total, 285 participants (226 female, 59 male; aged 19–26 years) completed the study. Participants were first-year Psychology students at the University of Groningen (Netherlands). No additional demographic information was collected, however, the cohort consisted primarily of Dutch and German students, along with other primarily European nationalities. Participants received course credit for participation.

### Design and procedure

**Figure 1:**
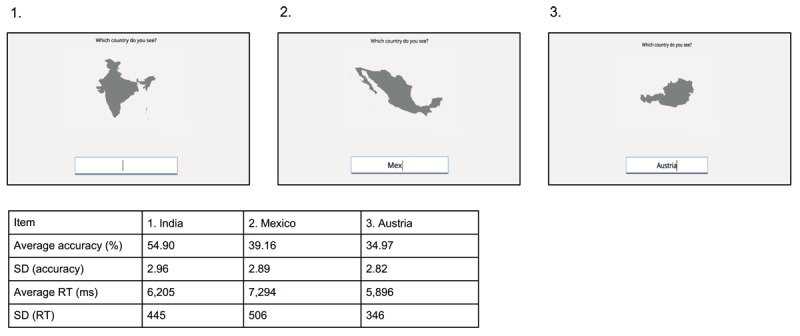
Country outlines were presented on the screen, and the participant was asked to type the correct name of the country in the text box. Normative accuracy and response time scores (see below for more details) of the three depicted countries are shown in the table below the figure.

As COVID-19 restrictions prevented any lab experiments, the study was conducted online using the jsPsych online experiment library (De Leeuw, 2015). The code to run the experiment is available at: https://osf.io/4kyz9. Participants were instructed to sit in a quiet room, and were asked to remain focused throughout the experiment. We programmatically checked whether participants clicked away or visited other web pages during the experiment, which did not occur in the reported study. Participants were instructed to attempt to name each country. Since we expected relatively low average performance, participants were explicitly encouraged to keep trying, even if they would not know the answer to several items in a row. Instructions were given in English, but participants were allowed to respond in Dutch or German as well. All trials had the same general structure (see Figure 1): A gray-filled country outline (see Materials) was presented on the screen, and participants were instructed to type its name in the text box. After the response, no feedback was given, and the next item was presented. If participants took longer than 25 seconds to respond, the next item was automatically presented. Participants cycled through all items once, in random order.

### Materials

This study used 112 country outline- name paired associates as stimuli. We used the 100 most populous countries,^1^ supplemented by 12 European countries that we thought participants were likely to know. The complete list can be found at: https://osf.io/d5qn9/.

The images used in this study were generated using R 3.4.1 (R Core Team, 2020), with packages ggplot2 (Wickham, 2016) and rnaturalearth (Andy, 2017). The script for generating the images can be found at: https://osf.io/rfv7b/. For some countries, the images were manually edited after they were generated. Outlines for some countries were manually cropped (for example, for the map of the Netherlands, the Netherlands Antilles were not included). The included code can be edited and extended to change, for example, the colors and size of the stimuli or the countries included in the set.

### Analysis

Analyses and data visualizations were performed in R 3.4.1 (R Core Team, 2020). For each country, different commonly-used names and abbreviations were considered correct responses. For example, for ‘United States of America’, alternatives such as ‘US’, ‘America’ and ‘USA’’ were considered correct responses (along with their Dutch and German equivalents). For a complete overview of alternative answers that were considered correct, see https://osf.io/d5qn9/. To prevent that (minor) typing errors would result in scoring a response as incorrect, responses were considered correct if *Levenshtein’s edit distance* from the response to the correct answer (i.e., the number of single digit edits required to change the response into the fully correct answer, see Yujian & Bo, 2007) was equal to or less than 2. Levenshtein’s edit distances were computed to all translations of the item. In situations in which the implementation of the Levenshtein’s edit distance could result in incorrect labels being scored as correct (e.g., when a participant responded ‘Iraq’ (instead of Iran) to the Iranian country outline, responses were manually checked. Data for one participant was excluded from the analysis, because of unrealistically fast average response times (242 ms) and low accuracy (0.0%).

## Dataset: Format

Two datasets are available on Open Science Framework. First, the **full_data** file contains raw data for all observations and is available in .csv and .rds format, see https://osf.io/q25rd/. Each row in the dataset represents a single trial in the experiment. Details about the column names can be found in Table 1.

**Table 1:** Column names and descriptions for the full dataset.


COLUMN	DESCRIPTION

rt	The response time in ms, measured by first key press (unless the backspace key was used, in which case we report an infinite response time (Inf))

trial_index	The index of the current trial

time_elapsed	The time elapsed in ms since the start of the experiment

participant_number	A unique id for each participant

id	A unique id for each country outline item

presentation_start_time	The start time of the trial, in ms elapsed since January 1, 1970, 00:00:00 UTC.

answer	The name of the presented country outline

correct	A binary accuracy of the response

backspace used	A logical variable indicating whether the backspace key was used in the response

response	The response entered by the participant

attempt	A binary variable indicating if the participant gave a response of at least two characters in length

rt_under_800	A variable indicating if the participant responded within 800 ms from stimulus presentation


Second, the **norms** file contains prior knowledge and response time norms for each country outline-name paired associate, see https://osf.io/uaq82. Items are sorted by ascending accuracy and descending response times. Details are listed in Table 2. The code to generate the latter summary from the **full_data** is also included.

**Table 2:** Column names and descriptions for the normative data file.


COLUMN	DESCRIPTION

answer	The name of the presented country outline

accuracy	The proportion of correct responses for the current item over all participants

accuracy_sd	The standard deviation for correctness on the current item over all participants

rt	The overall average response time for the current item over all participants in milliseconds (including response times for incorrect responses).

rt_sd	The standard deviation for response times for the current item over all participants in milliseconds


Next to average performance, the presented data allows for the calculation of various additional measures. For example, for each country, the number of correct responses, the number of incorrect responses, the type of incorrect response (i.e., null responses, swapped country names, partially retrieved country names, etc.) can be calculated.

## Dataset: overview

**Figure 2:**
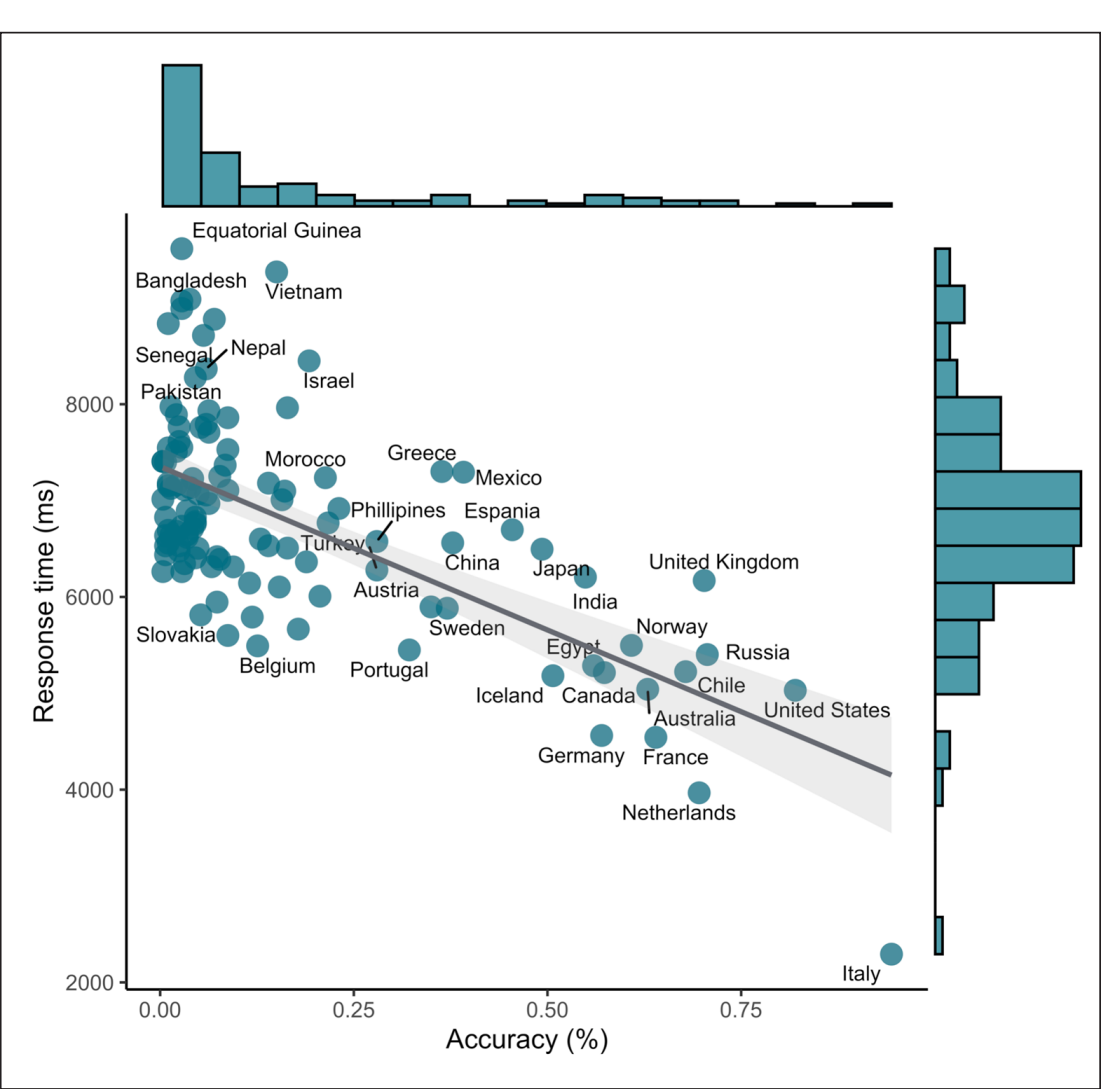
Association between average response times and accuracy. Marginal distributions of response times and accuracy are also shown. Points represent individual items, the gray line represents a linear model capturing the negative association between response times and accuracy scores. The shaded area represents a 95% confidence interval.

Figure 2 shows the association between average response times and average accuracy per item, as well as the distributions of accuracy and response times across items. Overall, 17% of all outlines were recognized correctly. Most items were recognized in less than 25% percent of all trials, and average correctness per item ranged from 0.3–94.4%. Only two items were recognized in more than 75% of all trials: *Italy* and the *United States of America*. The overall mean reaction time per item was 6,787 ms, and ranged from 2,290–9,610 ms. Figure 2 also shows that there is a negative relationship between accuracy and response times: Responses for countries with higher average accuracy (e.g., ‘Italy’, on the bottom-right) were usually faster than responses for countries with lower accuracy (e.g., ‘Equatorial Guinea’, top-left), (*r*(110) = –0.65, *p* < 0.001). We also found a negative correlation between response times on items that were *answered correctly* and average accuracy for these items, indicating that even among correct responses, response times could be used to differentiate between item difficulty/prior knowledge (*r*(110) = –0.36, *p* < 0.04).

**Figure 3:**
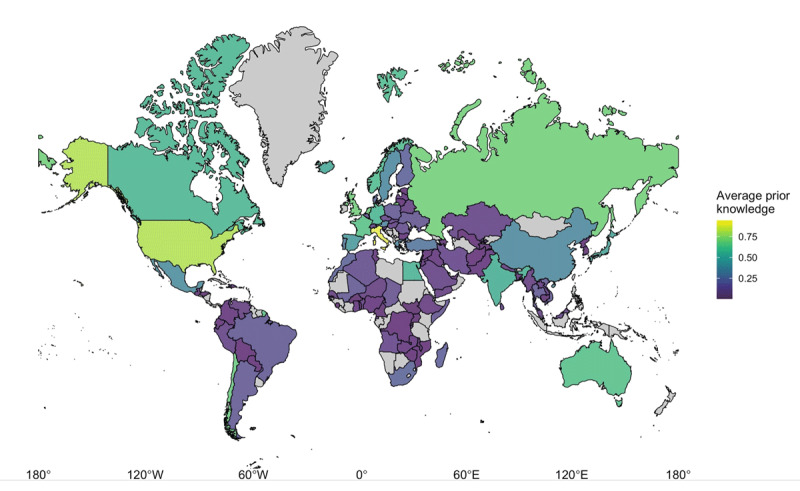
Average proportion of correct responses for each country. Uncolored countries were not presented in the current study. Note that this projection is landmass inaccurate: sizes away from the equator are inflated.

Figure 3 shows participants’ prior knowledge of country outlines on a world map. The figure shows that average accuracy varied across countries, but that no meaningful groups of countries for which participants showed similar performance stand out. In order to more formally inspect if there were specific groups of countries for which participants showed similar levels of prior knowledge, we performed a K-means cluster analysis on the data. The results did not reveal meaningful country clusters (e.g., groups by continent, see https://osf.io/u4597).

## Discussion

We present an open database containing prior knowledge norms for 112 country outline-name paired associate stimuli, providing a new tool for researchers interested in studying the mechanisms of learning in real-world situations where learners can be expected to have varying degrees of prior knowledge of learning materials. The stimulus set contains items for which participants had low, average, and high levels of prior knowledge, allowing researchers to select items with varying probabilities of prior knowledge.

Despite the fact that the presented data was collected in a carefully designed experiment conducted with a large group of participants, the results should be interpreted with some caution. First, only limited demographic information about the participants in this study is available. Second, for most items presented here, participants had low average prior knowledge, making it difficult to select a large item subset for which participants can be expected to have high prior knowledge. However, by providing response latencies for all items, we allow for meaningful differentiation of items with low prior knowledge. Furthermore, several adjustments of the task could be used to increase the average recognition accuracy, such as displaying the country scale and its position on the world map. Finally, although the current dataset includes responses from participants with different nationalities, they were all studying in the Netherlands at the moment of participation, and most participants had a Western European background. Therefore, the generalizability of the prior knowledge norms reported to participants living in other places in the world is unknown and should be examined in future research. Especially for research involving non-European participants, we recommend a new assessment of prior knowledge norms. However, we argue that a certain loss of generalizability is inherent to the type of materials presented. By nature, experiments that assume no prior knowledge are usually less dependent on the background of the learner. Moving to more applied settings and using materials that do assume prior knowledge ultimately involves a tradeoff between generalizability and the face validity of materials. Furthermore, as mentioned in the introduction, we provide all materials necessary to extend the current stimulus set with new materials, and test prior knowledge norms in new participant populations, which makes it possible to create a suitable stimulus set for participants with any nationality.

The materials presented here can be used in various types of research in the domain of learning declarative information. Specifically, they can be valuable in applied studies where conclusions about real-world learning are important. For example, in the growing field of computerized adaptive learning or cognitive tutoring (e.g., see Lindsey, Shroyer, Pashler, & Mozer, 2014; van Rijn, van Maanen, & van Woudenberg, 2009; Settles & Meeder, 2016), which aims to tailor the learning process to the needs of individual students, differentiating approaches based on prior knowledge may be important. More specifically, prior knowledge can be used to alleviate cold start problems in adaptive learning (Park, Joo, Cornillie, van der Maas, & Van den Noortgate, 2019; van der Velde, Sense, Borst, & van Rijn, 2021). Next to applied studies, the work presented here is relevant for several more fundamental areas of research. For example, in research in retrieval practice and attempted retrieval benefits, prior knowledge plays an important role (Arnold & McDermott, 2013; Koh, Lee, & Lim, 2018; Pastötter et al., 2022, and see Wilschut, Sense, van der Velde, & van Rijn, 2022, for a first application of the stimuli presented in this study).

In summary, we present a high quality database containing prior knowledge and response time norms for 112 country outline-name paired associates. These materials are ecologically valid, easily-extendable, language independent, and can be applied in a range of areas in learning research. Furthermore, as average prior knowledge ranged from zero to near perfect, our stimulus set allows for the selection of materials of which participants can be expected to have different degrees of prior knowledge. The presented stimulus set can provide a useful new tool for applied research in the field of learning and memory.

## Data accessibility statement

All materials, data and analysis scripts reported in this manuscript are publicly available at the Open Science Framework (https://osf.io/q25rd/).

## Footnotes

Based on https://worldpopulationreview.com/countries. Indonesia was not included because the high number of individual islands resulted in an unclear stimulus figure.
